# Dietary vitamin K is remodeled by gut microbiota and influences community composition

**DOI:** 10.1080/19490976.2021.1887721

**Published:** 2021-03-02

**Authors:** Jessie L. Ellis, J. Philip Karl, Angela M. Oliverio, Xueyan Fu, Jason W. Soares, Benjamin E. Wolfe, Christopher J. Hernandez, Joel B. Mason, Sarah L. Booth

**Affiliations:** a Jean Mayer USDA Human Nutrition Research Center on Aging, Tufts University, Boston, MA, USA; b The Friedman School of Nutrition Science & Policy, Tufts University, Boston, MA, USA; c Military Nutrition Division, US Army Research Institute of Environmental Medicine, Natick, MA, USA; d Department of Ecology and Evolutionary Biology, University of Colorado-Boulder, Boulder, CO, USA; e Soldier Effectiveness Directorate, US Army Combat Capabilities Developmental Command Soldier Center, Natick, MA, USA; f Department of Biology, Tufts University, Medford, MA, USA; g Schools of Mechanical and Aerospace Engineering & Biomedical Engineering, Cornell University, Ithaca, NY, USA

**Keywords:** Gut microbiota, micronutrient, vitamin K, menaquinone, stable isotope, metabolism

## Abstract

Vitamins have well-established roles in bacterial metabolism. Menaquinones (MKn, n = prenyl units in sidechain) are bacterially produced forms of vitamin K produced by the gut microbiota and consumed in the diet. Little is known about the influence of dietary vitamin K quinones on gut microbial composition and MKn production. Here, male and female C57BL6 mice were fed a vitamin K deficient diet or vitamin K sufficient diets containing phylloquinone (PK, plant-based vitamin K form), MK4, and/or MK9. DNA was extracted from cecal contents and 16S sequencing conducted to assess microbial composition. Cecal microbial community composition was significantly different in vitamin K deficient female mice compared to females on vitamin K sufficient diets (all *p* < .007). Parallel trends were seen in male mice, but were not statistically significant (all *p* > .05 but <0.1). Next, stable isotope-labeled vitamin K quinones were supplemented to male and female C57BL6 mice (^2^H_7_PK, ^13^C_11_MK4, ^2^H_7_MK7, ^2^H_7_MK9) and to an *in vitro* fermentation model inoculated with human stool (^2^H_7_PK, ^2^H_7_MK4, ^2^H_7_MK9, or vitamin K precursor ^2^H_8_-menadione). Vitamin K quinones in feces and culture aliquots were measured using LC-MS. *In vivo*, supplemented vitamin K quinones were remodeled to other MKn (^2^H_7_- or ^13^C_6_-labeled MK4, MK10, MK11, and MK12), but *in vitro* only the precursor ^2^H_8_-menadione was remodeled to ^2^H_7_MK4, ^2^H_7_MK9, ^2^H_7_MK10, and ^2^H_7_MK11. These results suggest that dietary vitamin K deficiency alters the gut microbial community composition. Further studies are needed to determine if menadione generated by host metabolism may serve as an intermediate in dietary vitamin K remodeling *in vivo*.

## Introduction

Diet is a known modulator of gut microbiota community composition, function, and metabolic activity.^1,2^ Presence or absence of individual macro- and micronutrients alters microbial composition and functional potential, and the gut microbiota, in turn, synthesizes, alters and metabolizes nutrients. Relative to macronutrients, comparatively less is known about the effects of micronutrients on gut microbiota composition and metabolic activity. However, bacteria have dependencies on vitamin and vitamin-derived cofactors for metabolism,^3^ and multiple micronutrients, including vitamin A, vitamin D, vitamin B12, folate, iron, magnesium, selenium, and zinc, have been found to impact the gut microbiota composition, particularly in deficiency.^2,4,5^ Micronutrients synthesized *de novo* by the microbiota also serve functional roles within the gut. For example, B-vitamin biosynthesis and “sharing” among the gut microbiota may be a potential stabilizer of the gut microbiome.^6,7^ Thus, micronutrient-microbiota interactions have implications for human health by modulating nutrient availability and bioactivity, and influencing community composition and metabolic activity of the gut microbiota.

**Figure 1.**
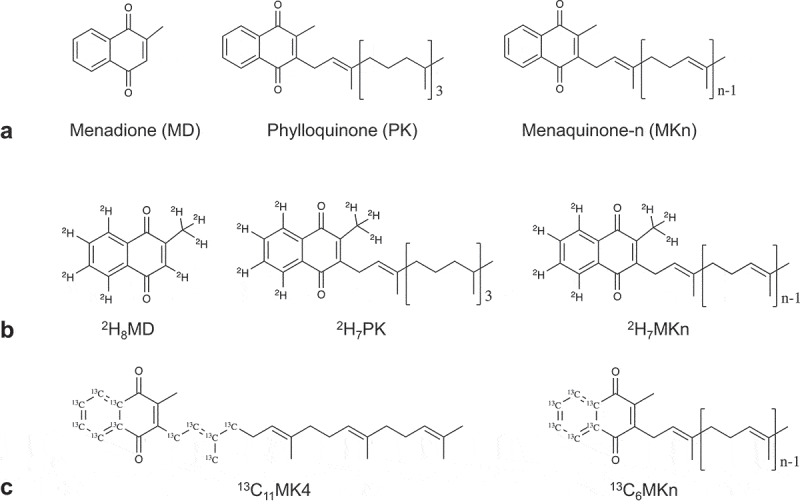
Chemical structures of vitamin K (VK) quinones. (a) All VK quinones share a common naphthoquinone ring (menadione (MD), considered a provitamin K) and can vary in length and saturation of the sidechain. Phylloquinone (PK) is produced by plants and has a mostly saturated sidechain, whereas menaquinones (MKn) are largely produced by bacteria and have unsaturated (n = number of prenyl units) sidechains. Stable-isotope label placement on **B**) deuterium (^2^H)-labeled and **C**) carbon-13 (^13^C)-labeled quinones used and/or detected in the stable isotope-labeled animal study (Study 2) and *in vitro* fermentation study (Study 3)

Vitamin K may be an underappreciated mediator of both diet-gut microbiota interactions and gut microbiota community dynamics. Whereas phylloquinone (PK) is a dietary vitamin K quinone found in plants,^8^ menaquinones (MKn) are vitamin K quinones that are both consumed in the diet and produced by the gut microbiota.^9^ All vitamin K quinones share a common naphthoquinone ring structure, but vary in the length and saturation of their sidechains (Figure 1a). MKn are used as electron carriers in bacterial respiration, and quinone synthesis is widespread in the gut.^10^ Some bacterial taxa have lost critical genes in MKn biosynthetic pathways, but still possess quinone-dependent terminal reductases, suggesting a retained capacity for respiration.^10^ Recent work demonstrated that a “growth factor” secreted from neighboring bacteria enabled the growth of previously uncultured human gut bacterial species, and the growth factor was identified to be MKn.^11^ Interestingly, although PK is not a bacterially produced vitamin K form, it has also been demonstrated *in vitro* to be a growth factor for some species of bacteria.^12,13^ These results together suggest some species in the human gut microbiota that have lost the ability to synthesize MKn may be able to utilize vitamin K quinones or precursors originating from neighboring bacteria or from the host diet. This would represent a tradeoff of a metabolically expensive synthesis pathway in favor of microbe-microbe or diet-microbe dependencies, which could, in turn, influence community stability and metabolic activity within the gut microbiota. However, to the best of our knowledge this concept has never been tested *in vivo*, nor has an intersection with host nutrition (specifically, dietary vitamin K quinones) been considered.

Evolved microbial dependencies are not without cost, and can lead to increased fragility of overall microbial community stability.^14^ Different bacterial taxa produce and utilize MKn of distinct lengths,^15,16^ though the relevance of sidechain length and reason for specificity are not entirely understood. A microbial capacity to remodel vitamin K quinones would buffer against dependence on specific quinones, the availability of which may dynamically change in the gut with shifts in microbial strains present or fluctuations in dietary supply. Conversely, an inability of gut microbial species to remodel vitamin K quinones presents a potential opportunity to use vitamin K quinones to target specific bacterial populations to modulate community composition, a strategy that has also been proposed for vitamin B12.^17^ Early work on bacterial vitamin K requirements indicated that bacteria may be able to metabolize one form of vitamin K to another,^13,18^ but this has never been systematically tested. Investigation of vitamin K-gut microbiota interactions would advance understanding of the role of bacterially derived MKn in human health, which is not currently well understood.

The objective of this study was to determine the influence of manipulating vitamin K (VK) intake on gut microbial community composition and MKn metabolism. To meet this objective, unlabeled and stable isotope-labeled VK quinones were supplemented in the diets of C57BL6 mice, and stable isotope-labeled VK quinones and a VK precursor (menadione, MD) were supplemented into an *in vitro* fermentation model inoculated with human stool. 16S sequencing of cecal samples and culture aliquots was completed to analyze the effect of VK supplementation on gut microbiota composition. Additionally, unlabeled and labeled VK quinones were measured in fecal samples and culture aliquots to investigate *de novo* MKn synthesis in the presence of supplemented vitamin K and possible remodeling of supplemented vitamin K forms by gut bacteria.

## Results

### Amount, more than specific form of dietary vitamin K quinone, influences murine cecal microbial community composition

Two vitamin K supplementation studies were conducted in C57BL6 mice utilizing representative dietary vitamin K quinones of varying sidechain saturation and length. In the first (Study 1), 50 male and 50 female mice were first placed on a vitamin K deficient diet (0.05 μmol PK/kg diet) for 4 weeks, and then randomized to continuation on vitamin K deficient diet or supplementation with 5.0 μmol/kg diet unlabeled vitamin K quinones (PK, MK4, MK9, or a combination PK/MK4/MK9) for 4 weeks (**Supplemental** Figure 1a). In the second (Study 2), 35 male and 35 female mice were first placed on a vitamin K sufficient diet (2.2 μmol unlabeled PK/kg diet) for 6 weeks, and then randomized to remain on the control vitamin K sufficient diet (unlabeled PK) or supplementation with an equimolar amount (2.2 μmol/kg diet) of stable isotope-labeled vitamin K quinones (^2^H_7_PK, ^13^C_11_MK4, ^2^H_7_MK7, ^2^H_7_MK9; label placement shown in Figure 1b-c) for 1 week (Supplemental Figure 1b). At sacrifice, cecal contents were collected for DNA extraction and 16S sequencing, and feces were collected for analysis of vitamin K quinone content.

**Figure 2.**
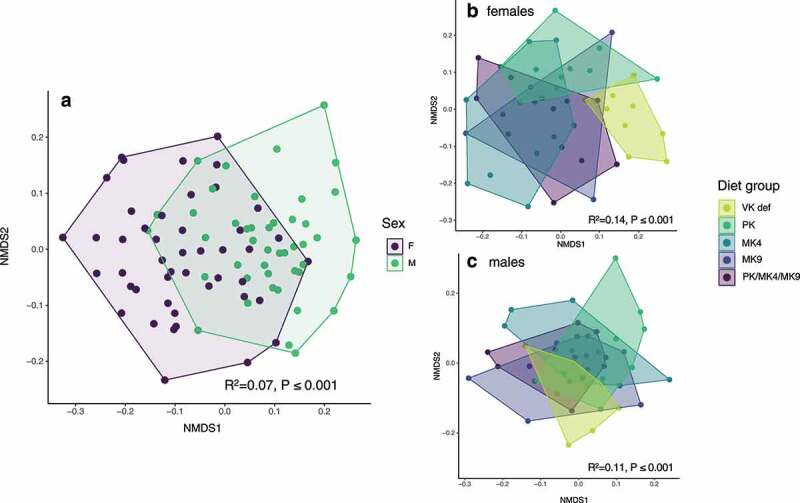
Non-metric multidimensional scaling (NMDS) ordination of mouse cecal microbial communities in the unlabeled vitamin K supplementation study (Study 1). (a) Male and female mice had significantly different cecal microbial communities. Microbial communities were also significantly different by diet in both (b) female and (c) male mice. PK = phylloquinone, MK4 = menaquinone-4, MK9 = menaquinone-9, VK def = vitamin K deficient

In Study 1, cecal microbial community composition at sacrifice significantly varied by sex (Figure 2a) and diet (overall PERMANOVA r^2^ = 0.013 and *P* < .001). When stratified by sex, diet was a significant predictor of community composition in both female and male mice (Figure 2b,Figure 2c). Within females, microbiota composition in the vitamin K deficient group differed significantly from supplemented groups (for all, *P* < .01). The PK group was also significantly different from the MK4 and MK9 groups (*P* < .01 for both). For male mice, the vitamin K deficient group trended toward being different from all supplemented groups and the PK group trended toward being different from the MK4 group and the PK/MK4/MK9 group (all *P* < .1 but >0.05 after corrections for multiple comparisons). Shannon diversity was significantly greater in female mice as compared to males (r^2^ = 0.16, *P* < .001) and did not differ by diet group (data not shown).

**Figure 3.**
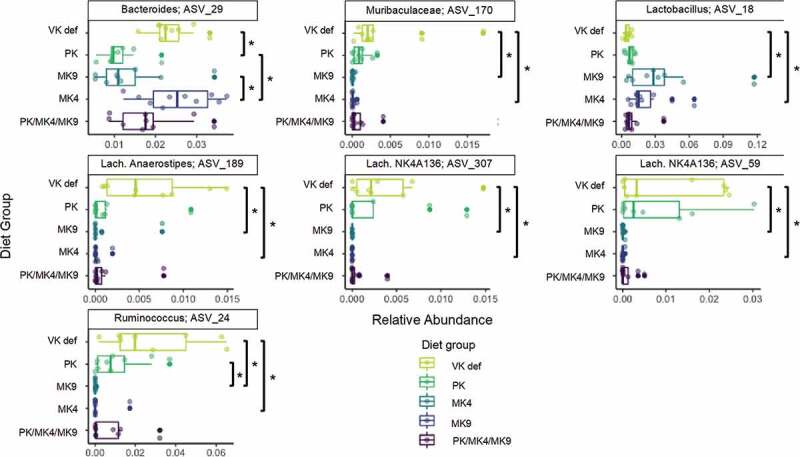
Microbial taxa that were significantly enriched across different diet groups in female mice of the unlabeled vitamin K animal study (Study 1, FDR-corrected p ≤ 0.05). Assessed with Kruskal-Wallis tests across diet groups for all ASVs with a mean abundance threshold of 0.001 (the minimum mean value needed in at least one of the factor levels for an ASV to be retained in the analysis). *indicates a pairwise comparison *p* < .05. No taxa were significantly enriched across diet groups in male mice after FDR adjustment. PK = phylloquinone, MK4 = menaquinone-4, MK9 = menaquinone-9, VK def = vitamin K deficient

Relative abundance of several bacterial taxa significantly (FDR *P* < .05) differed across diet groups in females (Figure 3). A *Ruminococcus* ASV, a *Lachnospiraceae Anerostipes* ASV, two *Lachnospiraceae* NK4A136 group ASVs, and a *Muribaculaceae* ASV were enriched in the vitamin K deficient group, whereas a *Bacteroides* ASV was enriched in the MK4 group, and a *Lactobacillus* ASV in the MK9 group. At the genus level, several genera were marginally (FDR *P* < .1) different by diet in female mice (**Supplemental Figure 2**): the vitamin K deficient group had the lowest relative abundance of *Lactobacillus*, and the greatest relative abundances of *Bacteroides* and a *Ruminococcus* genus group (*Ruminococcus_1*). No bacterial taxa or genera significantly differed across diet groups in male mice.

A sensitivity analysis was conducted removing the vitamin K deficient group. When removed, the percent variability explained by sex increased (r^2^ = 0.066 to r^2^ = 0.083). The overall effect of diet on microbiota composition in females was still significant (PERMANOVA r^2^ = 0.13, *P* = .01), with the MK4 and MK9 groups significantly different from the PK group (both *P* < .01), however, the relative abundance of no specific taxa or genera differed by diet group after FDR-*p*-value correction. In males, the effect of diet on microbiota composition was no longer significant (PERMANOVA r^2^ = 0.10, *P* = .052).

In Study 2 (in which all diets were vitamin K sufficient), the cecal microbial community composition at sacrifice was significantly different by sex (PERMANOVA r^2^ = 0.38 and *P* < .001, **Supplemental** Figure 3a), but did not differ by diet in either female or male mice (Supplemental Figure 3b and 3c). No differences were observed in composition between unlabeled (control) and stable-isotope labeled diets that would suggest an influence of the stable isotope labeling, though this cannot be entirely ruled out. Shannon diversity was again significantly greater in female mice as compared to males (r^2^ = 0.49, *P* < .001), but did not statistically differ by diet group (data not shown).

### Supplemented vitamin K quinones are remodeled by murine gut microbes

In Study 1, with supplementation of unlabeled vitamin K quinones, fecal vitamin K content reflected the form(s) supplemented in the diet. Endogenously produced MKn could not be differentiated from dietary MK4 and MK9. However, the only vitamin K forms in feces that statistically differed by group were those supplemented in the diets (PK, MK4, and MK9, Table 1). When forms used in the diet were excluded, there was no significant difference in any of the remaining fecal VK forms (MK5-MK8, MK10-MK13) by diet group.

**Table 1.** Fecal data from unlabeled animal study (Study 1)

	MALES					FEMALES					2-way ANOVA p-values		
Feces	*Diet group*					*Diet group*					**Sex**	**Group**	Sex*Group interaction
**Vitamer**	**VK def**	**PK**	**MK4**	**MK9**	**Combo**	**VK def**	**PK**	**MK4**	**MK9**	**Combo**	-	-	-
**PK**pmol/g	ND	2071^a^ ± 146	ND	ND	691^b^ ± 40.0	ND	2724^c^ ± 121	ND	ND	915^d^ ± 48.8	0.002	<0.001	0.96
MK4*pmol/g	1374^a^ ± 122	1617^a,b^ ± 176	2054^c^ ± 109	1844^a,b,c^ ± 194	2024^b,c^ ± 269	1575^a^ ± 107	1616^a,b^ ± 128	2161^c^ ± 126	1694^a,b,c^ ± 107	2055^b,c^ ± 250	0.70	<0.001	0.83
**MK5**pmol/g	6.1 ± 0.8	6.2 ± 1.0	6.5 ± 1.3	7.6 ± 1.0	6.4 ± 0.8	7.9 ± 1.5	10.4 ± 1.4	8.6 ± 1.2	8.7 ± 1.3	8.8 ± 1.7	0.001	0.23	0.63
**MK6**pmol/g	828 ± 210	562 ± 107	601 ± 105	636 ± 103	561 ± 113	679 ± 143	798 ± 112	590 ± 122	535 ± 122	624 ± 135	0.89	0.62	0.56
**MK7**pmol/g	30.9 ± 11.7	43.8 ± 16.2	44.9 ± 35.9	29.2 ± 6.7	27.8 ± 11.5	70.2 ± 28.9	89.5 ± 16.0	91.2 ± 16.6	63.4 ± 8.3	91.9 ± 20.8	<0.001	0.26	0.79
**MK8**pmol/g	174 ± 32.2	221 ± 37.1	224 ± 31.7	238 ± 39.0	190 ± 26.2	325 ± 55.6	362 ± 44.2	305 ± 48.3	346 ± 45.2	349 ± 57.1	<0.001	0.78	0.82
**MK9**pmol/g	213^a^ ± 56.4	287^a^ ± 37.0	275^a^ ± 53.8	3459^b^ ± 434	990^c^ ± 117	344^d^ ± 84.5	421^d^ ± 52.0	310^d^ ± 71.8	5355^e^ ± 499	1884^f^ ± 187	<0.001	<0.001	0.68
**MK10**pmol/g	938 ± 172	1167 ± 201	1137 ± 181	1360 ± 332	1302 ± 146	1237 ± 196	1566 ± 161	1394 ± 240	1664 ± 208	1524 ± 198	0.02	0.28	0.99
**MK11**pmol/g	534 ± 101	676 ± 93.6	632 ± 106	712 ± 137	645 ± 56.1	716 ± 115	883 ± 77.3	763 ± 169	856 ± 102	786 ± 91.9	0.02	0.54	0.99
**MK12**pmol/g	67.8 ± 24.6	77.3 ± 12.1	92.2 ± 19.7	97.4 ± 19.4	87.3 ± 9.8	61.4 ± 11.9	89.3 ± 13.3	93.1 ± 28.5	98.5 ± 17.6	88.0 ± 11.1	0.89	0.15	0.98
**MK13**pmol/g	196 ± 84.7	150 ± 34.1	255 ± 83.6	204 ± 79.2	179 ± 54.3	59.9 ± 62.6	99.6 ± 50.9	107 ± 57.0	130 ± 39.8	91.2 ± 37.6	<0.001	0.56	0.72

ND – nondetectable (limits of detection: PK and MK4, 30 pmol/g; MK6, 10 pmol/g; MK5, MK7-MK9, MK11-13, 5 pmol/g; MK10, 1 pmol/g).

*Some interference on the MK4 ion, therefore these concentrations should not be interpreted as absolute or used for future power or sample size calculations (these data were left in table for conceptual value only – for relative comparison to other groups – to show that fecal VK content reflected the form found in the diet). For the second animal study (Table 2), fecal samples were additionally run on a C30 column to separate out the MK4 ions from interference.

**Table 2.** Fecal data from stable isotope animal study (Study 2)

		**MALES**					**FEMALES**					**2-way ANOVA p-values**		
		*Diet group*					*Diet group*					**Sex**	**Group**	Sex*Group
*Classification*	*pmol/g*	**Control (unlabeled PK)** n=7	**^2^H_7_PK** n=7	**^13^C_11_MK4** n=7	**^2^H_7_MK7** n=7	**^2^H_7_MK9** n=7	**Control (unlabeled PK)** n=6	**^2^H_7_PK** n=7	**^13^C_11_MK4** n=7	**^2^H_7_MK7** n=7	**^2^H_7_MK9** n=7	**^−^**	**^−^**	**^−^**
**Supplemented form**	PK	1919 ± 93.8	ND	ND	ND	ND	2450 ± 232	ND	ND	ND	ND	0.05	-	-
	^2^H_7_PK	ND	485 ± 76.3	ND	ND	ND	ND	674 ± 89.7	ND	ND	ND	0.11	-	-
	^13^C_11_MK4	ND	ND	2133 ± 164	ND	ND	ND	ND	2234 ± 102	ND	ND	0.61	-	-
	^2^H_7_MK7	ND	ND	ND	1739 ± 92.2	ND	ND	ND	ND	2229 ± 290	ND	0.08	-	-
	^2^H_7_MK9	ND	ND	ND	ND	3089 ± 250	ND	ND	ND	ND	4512 ± 700	0.07	-	-
**Unlabeled forms**	MK4	ND*	ND*	ND*	ND*	ND*	44.3 ± 9.1	ND*	ND*	ND*	ND*	0.006	-	-
	MK5	14.7 ± 5.5	8.6 ± 6.0	4.3 ± 2.8	6.2 ± 3.0	5.2 ± 2.0	17.7 ± 4.1	10.9 ± 0.9	6.9 ± 3.7	10.5 ± 1.8	10.7 ± 2.8	0.48	0.23	0.98
	MK6	903 ± 163	649 ± 212	708 ± 114	659 ± 123	692 ± 170	1348 ± 321	1239 ± 176	1207 ± 358	1285 ± 215	1153 ± 207	<0.001	0.77	0.96
	MK7	47.5 ± 19.3	66.5 ± 149	29.8 ± 18.1	54.3 ± 50.8	25.7 ± 2.8	118 ± 33.6	65.0 ± 14.7	74.3 ± 63.3	66.6 ± 13.1	63.1 ± 13.4	0.005	0.29	0.37
	MK8	187 ± 25.6	240 ± 59.1	208 ± 20.3	212 ± 32.2	210 ± 23.4	330 ± 53.1	263 ± 16.8	301 ± 37.7	305 ± 29.4	277 ± 27.3	<0.001	0.99	0.45
	MK9	322 ± 28.7	327 ± 57.3	341 ± 54.4	321 ± 50.2	299 ± 55.1	423 ± 43.7	359 ± 32.0	403 ± 48.4	414 ± 49.7	365 ± 33.3	0.01	0.83	0.95
	MK10	622 ± 73.9	564 ± 78.2	535 ± 90.8	471 ± 73.9	440 ± 82.5	990 ± 132	806 ± 36.9	888 ± 103	839 ± 79.4	769 ± 67.1	<0.001	0.17	0.91
	MK11	745^a^ ± 148	420^b^ ± 53.2	390^b^ ± 68.2	335^b^ ± 40.6	297^b^ ± 57.7	937^c^ ± 126	679^d^ ± 27.5	770^d^ ± 77.2	743^d^ ± 93.5	621^d^ ± 60.9	<0.001	<0.001	0.18
	MK12	325^a^ ± 106	155^a,b^ ± 32.3	138^b^ ± 23.5	114^b^ ± 11.4	93.7^b^ ± 25.7	359^c^ ± 47.3	269^c^ ± 18.4	315^c^ ± 29.8	301^c^ ± 35.0	232^c^ ± 33.7	<0.001	<0.001	0.05
	MK13	1.4 ± 11.5	0.2 ± 2.8	0.2 ± 6.2	0.2 ± 7.4	ND	ND	0.2 ± 4.8	ND	ND	ND	0.06	0.35	0.30
**Labeled forms**	^2^H_7_MK4/^13^C_6_MK4	ND^a^	12.0^b^ ± 2.0	8.5^b^ ± 1.0	10.4^b^ ± 1.8	13.4^b^ ± 1.2	ND^c^	68.2^d^ ± 6.5	43.4^e^ ± 4.3	68.5^d^ ± 6.1	59.2^d,e^ ± 6.8	<0.001	<0.001	<0.001
	^2^H_7_MK10/^13^C_6_MK10	ND^a^	46.9^b^ ± 7.4	59.1^b^ ± 8.4	57.6^b^ ± 10.4	61.8^b^ ± 10.0	ND^c^	113^d^ ± 18.9	152^d^ ± 21.9	137^d^ ± 45.1	166^d^ ± 28.2	<0.001	<0.001	0.02
	^2^H_7_MK11/^13^C_6_MK11	ND^a^	209^b^ ± 21.3	335^c^ ± 49.4	255^b,c^ ± 36.1	218^b,c^ ± 41.1	ND^a^	194^b^ ± 31.5	312^c^ ± 37.0	223^b,c^ ± 38.7	252^b,c^ ± 38.7	0.76	<0.001	0.90
	^2^H_7_MK12/^13^C_6_MK12	ND^a^	95.0^b^ ± 12.6	158^c^ ± 15.0	121^b,c^ ± 23.7	110^b,c^ ± 17.7	ND^d^	70.5^e^ ± 8.5	106^f^ ± 9.6	76.6^e,f^ ± 14.3	78.7^e,f^ ± 11.8	<0.001	<0.001	0.43

Geometric mean ± SEM. ND – nondetectable (limits of detection: PK and MK4, 30 pmol/g; MK6, 10 pmol/g; MK5, MK7-MK9, MK11-13, 5 pmol/g; MK10, 1 pmol/g). * = trace amounts unlabeled MK4 detected. Some trace ^2^H_7_MK9/^13^C_6_MK9 also detected, but in less than 20% of animals, and so data were not included.

**Figure 4.**
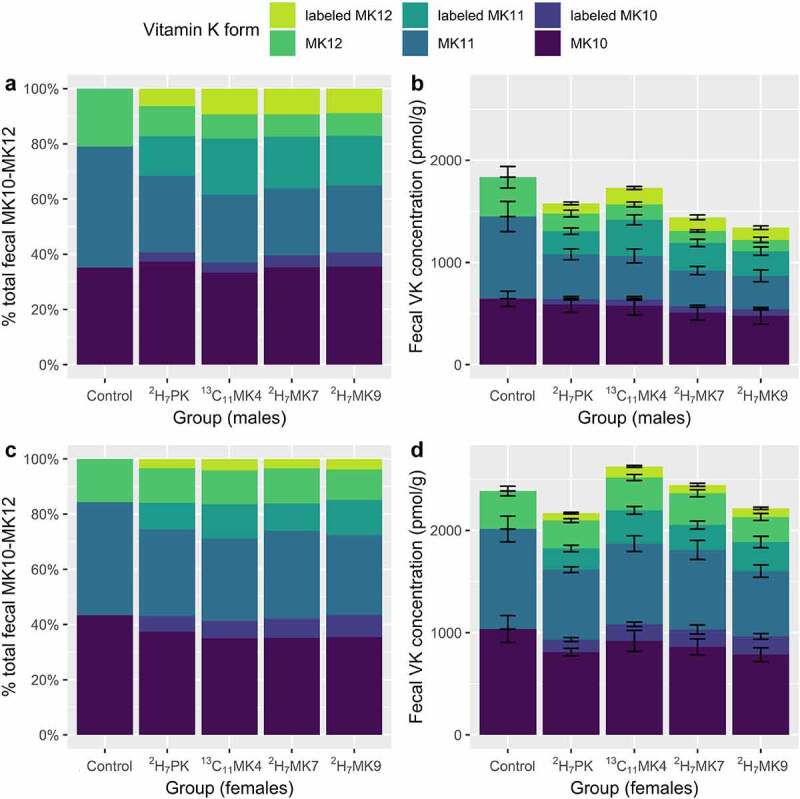
Unlabeled and stable isotope labeled (^2^H_7_- or ^13^C_6_-) MK10-MK12, shown as mean % total MK10-MK12 (a and c) and mean concentrations (b and d), in male and female mice in the labeled animal study (Study 2). Error bars on the stacked bar charts represent the standard error of the mean. Unlabeled and stable isotope-labeled MK4 were excluded from the analysis because MK4 deriving from host intestinal conversion could not be differentiated from bacterially produced MK4

In Study 2, the labeling on the supplemented stable isotope-labeled quinones allowed differentiation of dietary from endogenous vitamin K quinones. In addition to the stable isotope forms supplemented in the diet, isotopically labeled (^2^H_7_ or ^13^C_6_) MK4, MK10, MK11, and MK12 were detected in feces of stable isotope-supplemented mice (Table 2). Females had higher total (unlabeled + labeled) MK10-MK12 than males (*P* < .001), but within sex there were no differences between diet groups (*P* = .32, Figure 4). Total endogenous fecal VK (excluding dietary forms) was not different by diet group (*P* = .40).

### Human gut microbes remodel a vitamin K precursor, but not full vitamin K quinones, to other menaquinones in vitro

A third study (Study 3) was conducted *in vitro* using human gut microbes. Stool from 5 healthy male donors was pooled and inoculated in bioreactors under conditions mimicking the human colon. Individual bioreactors were designated as controls or treated with stable isotope-labeled VK quinones (^2^H_7_PK, ^2^H_7_MK4, ^2^H_7_MK9) or the provitamin K form menadione (^2^H_8_MD) and fermented for 48 h under constant nitrogen flow (Supplemental Figure 1c). Aliquots were collected at 0, 5, 10, 24, and 48 hours. DNA and vitamin K quinones were extracted from aliquots for 16S sequencing and LC-MS quantification, respectively. The experiment was conducted in triplicate.

**Figure 5.**
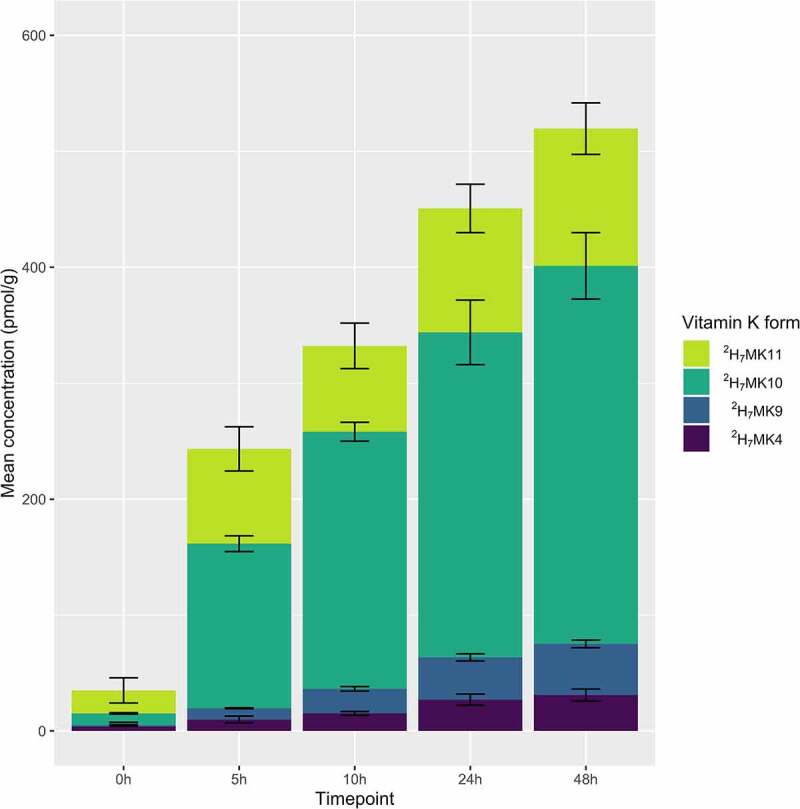
Production of ^2^H-labeled MKn at each time point in the ^2^H-MD supplemented vessel in Study 3. ^2^H-labeled MKn were not recovered in any of the other supplemented vessels. Stacked bars represent the mean (n = 3 experiments) concentrations, with error bars representing standard deviation from the mean. ^2^H_7_MK11 concentrations are corrected for interference

Endogenous (unlabeled) production of MKn was unaffected by supplementation of ^2^H-quinones (**Supplemental Figure 4**). Remodeled quinones (^2^H_7_MK4, ^2^H_7_MK9, ^2^H_7_MK10, and ^2^H_7_MK11) were detected in ^2^H_8_MD supplemented vessels and accumulated over time (Figure 5), but were not detected in controls or ^2^H_7_PK, ^2^H_7_MK4, or ^2^H_7_MK9 supplemented vessels.

Microbial community composition was analyzed at 0 h and 24 h timepoints. Communities significantly differed by timepoint (PERMANOVA r^2^ = 0.86 and *p* < .001), but not by vitamin K quinone treatment (even when analyzed at 24 h only, **Supplemental Figure 5**).

### Murine cecal bacterial genera enrichment and fecal vitamin K quinone content differ by sex

In both animal supplementation studies, genera *Lachnospiraceae* FCS020 group, *Roseburia*, and *Ruminococcaceae* UCG-009 were significantly enriched in female mice as compared to males, and *Ruminococcus_1* was significantly enriched in male mice as compared to females (**Supplemental Tables 1 and 2**). The same relationships held when the vitamin K deficient groups were removed in the first study, with an additional *Parabacteroides* genus enriched in males (**Supplemental Table 3**).

Sex effects were observed for most fecal vitamin K forms. In Study 1, female mice had greater fecal concentrations of PK, MK5, and MK7-MK11, and male mice had greater fecal concentrations of MK13 (Table 1). In Study 2, female mice had greater fecal concentrations of the supplemented dietary PK, endogenous unlabeled MK4 and MK6-MK12, and remodeled stable isotope-labeled MK4 and MK10. Male mice had greater remodeled stable isotope-labeled MK12, and demonstrated a trend (*p* = .06) for greater unlabeled MK13 (Table 2).

## Discussion

The work described herein included study of microbial community composition in both vitamin K deficient and vitamin K sufficient *in vivo* models, and study of MKn metabolism using stable isotopes in both *in vitro* and *in vivo* models. This multi-faceted approach provided complementary insights into vitamin K-gut microbiota interactions, and resulted in two novel findings: 1) dietary vitamin K amount influences microbial community composition, an effect that appears more pronounced in murine females than males, and 2) vitamin K quinones are remodeled *in vivo*.

In Study 1, gut microbiota community composition was significantly different in vitamin K deficient as compared to all vitamin K sufficient groups of female C57BL6 mice. Parallel trends were seen in male mice, but were not significant (*p* > .05 but <0.1 for all). In Study 2, all diets were vitamin K sufficient, and no differences in gut microbiota composition were seen by supplemented vitamin K form in either female or male mice. Differences by supplemented vitamin K form were seen only in females in Study 1, which were supplemented at a higher dose and for a longer duration than in Study 2. Taken together, these results suggest that the *amount* of vitamin K, more so than the specific form, influences the composition of the murine cecal microbiota.

Four of the five taxa enriched in vitamin K deficient females were within the Lachnospiraceae and Ruminococcaceae families (both within Clostridiales order). Neither appear to be menaquinone producers,^19^ and both families are butyrate producers,^20^ suggesting their main form of energy production is likely saccharolytic fermentation, not anaerobic respiration. This may suggest that low availability of exogenous vitamin K allowed non-MKn utilizing microbes to outcompete those that require MKn. Similarly, bacterial taxa that cannot adequately synthesize MKn, but can utilize MKn for respiration, may also be outcompeted when exogenous vitamin K availability is low. Vitamin K deficient females had lower relative abundance of a *Lactobacillus* ASV as compared to females supplemented MK4 or MK9. Lactobacillales are believed to have undergone massive gene losses, including loss of MKn synthesis genes, yet some are still able to respire when heme and MKn are present.^21^ Collectively, these findings are consistent with reports of alterations in gut microbiota composition with other micronutrient deficiencies,^22^ and supports the concept that availability of dietary micronutrients impacts competitive dynamics within the gut microbiota.

All stable isotope-labeled VK forms supplemented *in vivo* (^2^H_7_PK, ^13^C_11_MK4, ^2^H_7_MK7, and ^2^H_7_MK9) were remodeled to labeled MK4, 10, 11 and 12 in feces, and in similar concentrations across diet groups. This suggests a common remodeling mechanism. That hypothesis was supported by the fact that the stable isotope labeling on the naphthoquinone ring was preserved. All mice supplemented quinones with ^2^H_7_-labeling on the ring retained the ring labeling on the remodeled quinones detected in feces. ^13^C-labeling was present on both the ring and sidechain of the supplemented ^13^C_11_MK4, but only ^13^C_6_-labeled MKn (only on the ring, see Figure 1c) were detected in feces. This indicates complete sidechain replacement, and is suggestive of MD (the naphthoquinone ring) as the intermediate in the remodeling.

*In vitro*, only ^2^H_8_MD, but not full VK quinones, were remodeled to ^2^H_7_MKn. These results suggest that human gut bacteria cannot remove the sidechain of VK quinones, but can add a sidechain to a vitamin K precursor. A comparison of *in vitro* and *in vivo* models here suggests that there may be a keystone species in the murine, but not human, gut microbiota that can cleave the naphthoquinone ring from the sidechain, or that the host may generate a necessary intermediate in the remodeling of dietary vitamin K forms. MD is one possible candidate, as it is generated in intestinal tissue during the enzymatic conversion of dietary VK to MK4. The reaction is independent of the gut microbiota,^23^ and involves prenylation of MD by the mammalian enzyme UBIAD1.^24,25^ As enterocytes are routinely sloughed off every 2–3 days,^26^ host-generated MD may become available to the gut microbiota upon enterocyte shedding. MenA is a prenyltransferase in one of the two *de novo* bacterial synthesis pathways of MKn,^10^ and is the bacterial homolog of UBIAD1. Therefore, MenA is a likely candidate for the prenyltransferase involved in the remodeling of dietary vitamin K quinones. Although we have demonstrated *in vitro* the capability of human gut microbiota to prenylate MD to form menaquinones, further experiments are needed to confirm if MD serves as an intermediate *in vivo*.

The results presented here nonetheless suggest MKn may be an important commensal factor in the gut, presenting a potentially novel role for vitamin K as a modulator of gut microbial composition. The relevance of gut bacterially produced menaquinones to human health (at least for known functions of vitamin K) has long been speculative.^27^ In the host, vitamin K functions as a cofactor for the carboxylation of vitamin K-dependent proteins involved in diverse functions such as blood clotting and regulation of calcification.^28^ Historically, gut bacteria were thought to contribute up to 50% of the vitamin K requirement of the host. However, there is no known mechanism of vitamin K absorption from the colon,^29^ and cofactor activity of vitamin K decreases with increasing sidechain length.^30^ Furthermore, vitamin K inadequacy in the host is created when vitamin K is removed from the diet,^31^ implying that the majority of the human vitamin K requirement comes from the diet. Gut bacterially produced MKn may instead contribute indirectly to human health through modulation of gut microbiota composition, and our results suggest that dietary vitamin K may be an influential factor in these dynamics. Vitamin B12 has similarly been demonstrated to be remodeled by gut bacteria and has been proposed to be a modulator of gut microbial ecology.^32^ Collectively, these data further the understanding of micronutrient-microbiota interactions.

Strengths of this study include the use of stable isotopes to trace the metabolism of supplemented vitamin K quinones and differentiate remodeled MKn from those synthesized *de novo*. Additionally, the use of complementary *in vivo* and *in vitro* models allowed for the discovery of a potential host-gut microbiota interaction in the metabolism of MKn. The inclusion of both male and female mice here is also a strength which revealed differences in microbial community composition by sex, consistent with previous reports of sex differences in C57BL6 mice.^33,34^ Interestingly, sex differences were observed in the murine gut microbiota response to dietary vitamin K, with cecal microbial communities in females appearing more responsive to dietary vitamin K manipulation at higher doses in Study 1. Fecal MKn also differed by sex, consistent with previous reporting from our lab.^35^ Many genera that differed between male and female mice were within the Lachnospiraceae and Ruminococcaceae families, which are not known to be MKn producers. Therefore, differences in community composition do not appear to explain sex differences in fecal MKn. However, bacterial load is higher in male as compared to female C57BL6 mice.^36^ This suggests differences in absolute abundance that we could not detect, or a host component (such as hormonal or sex chromosomal factors), may be responsible for observed sex differences in fecal MKn.

Findings should also be interpreted within the context of several limitations. The sequencing method did not allow us to investigate absolute abundance or more granular species-level differences in bacterial communities or gene content. It is plausible that the acquisition or partial remodeling of supplemented vitamin K quinones could allow for diversion of energetic resources, normally used for MKn synthesis, to other functions. Additionally, as the microbiome varies across gastrointestinal regions,^37^ measurement of luminal vitamin K in conjunction with metagenomic sequencing would be informative in understanding the locale and predominant bacterial taxa associated with vitamin K remodeling, and the possible relevance for host vitamin K status. Furthermore, although the plausibility of MD as the proposed intermediate in the remodeling of dietary vitamin K quinones by gut bacteria was demonstrated *in vitro* with ^2^H_8_MD, we did not quantify MD *in vivo*. MD is highly reactive, and to our knowledge, there is no existing assay that can directly measure the compound in tissue or feces. Future studies should focus on investigation into functional and strain-specific usage of vitamin K in the gut using metagenomic sequencing and measurement of MD. Finally, along with the limitations of applying *in vitro* results to *in vivo* systems, a scenario in which exogenous (supplemented or dietary) vitamin K is present in the gut at higher concentrations than endogenously produced MKn is unlikely but not well studied. As this was the case in the *in vitro* study, results should be interpreted as proof-of-concept demonstration that human-associated gut microbes have the capability to partially metabolize exogenous vitamin K. Further investigation of that capability, and human *in vivo* research to determine the extent to which dietary and supplemental vitamin K may be remodeled to bacterial menaquinones in the human gut, is warranted.

In conclusion, dietary vitamin K amount, more so than the specific form, influences murine gut microbial community composition. Dietary vitamin K is remodeled to bacterial menaquinones by murine gut microbes *in vivo*, and vitamin K precursor MD (but not full vitamin K forms) are remodeled to bacterial menaquinones by isolated human gut microbes *in vitro*. Further studies are needed to determine if MD generated by host metabolism may serve as an intermediate in dietary vitamin K remodeling. These findings add vitamin K to a growing list of dietary micronutrients that appear to influence gut microbiota composition and metabolic activity.

## Materials and methods

### Animal models

Male and female C57BL6 mice were purchased from Charles River Laboratories (Wilmington, MA). All animal experiments and protocols were approved by the Institutional Animal Care and Use Committee at the Human Nutrition Research Center on Aging at Tufts University.

### Materials

Purified VK forms were obtained from Sigma-Alderich (PK, ^2^H_7_PK, MK4, ^2^H_7_MK4, MK9, ^2^H_7_MK9, and ^2^H_8_MD; St. Louis, MO), IsoSciences (^13^C_11_MK4, ^2^H_7_MK7, and ^2^H_7_MK9; Ambler, PA), and GL Synthesis (K_1(25)_; Worcester, MA). All HPLC-grade solvents (Fisher Scientific Inc., Springfield, NJ) were used for extraction and chromatography procedures described below.

### Preparation of animal study diets

All animal diets were mixed on-site to customize and ensure diet VK concentrations. Purified VK forms were first solubilized in tocopherol-stripped corn oil (5%, CA.160160, Envigo), and then mixed into a vitamin K deficient basal diet mix (95%, TD.120060, Envigo). The basal mix contained no menadione (MD), which is a pre-vitamin often used as a source of vitamin K in rodent chow. PK, MK4, MK7, and MK9 were chosen as representative vitamin K forms of varying saturation and side chain length. These forms are also present in the human diet, with PK abundant in plants, MK4 in animal meats, MK7 in natto (a commonly consumed fermented soybean product in Japan) and popular as a dietary supplement, and MK9 in dairy products.^8^ The recommended dietary vitamin K content of rodent diet is 1.0 mg PK/kg diet (molar equivalent 2.2 μmol PK/kg),^38^ and this is the level to which the diets were formulated in Study 2 to represent physiologically relevant concentrations for a C57BL6 mouse. Study 1 diets were formulated at a little more than double this concentration (5.0 μmol/kg) to represent a supplementation scenario.

### Animal study supplementing unlabeled VK quinones (Study 1)

Fifty male and 50 female 10-week old C57BL6 mice were first placed on a vitamin K-deficient diet (0.05 μmol/kg PK) for 4 weeks, after which they were then randomized to five groups: maintenance on the vitamin K-deficient diet, or placed on a supplemented diet containing 5 μmol/kg PK, MK4, MK9, or an equimolar combination of PK/MK4/MK9, for 4 weeks (Supplemental Figure 1a). Mice were 18 weeks of age at sacrifice, at which time cecal contents and feces were collected.

### Animal study supplementing stable isotope-labeled quinones (Study 2)

Thirty-five male and 35 female 12-week old C57BL6 mice were first acclimated on a vitamin K-sufficient (2.2 μmol/kg unlabeled PK) diet for 6 weeks, and then randomized to five groups: maintenance on unlabeled PK diet, or a diet containing 2.2 μmol/kg stable isotopically labeled vitamin K forms: ^2^H_7_PK, ^13^C_11_MK4, ^2^H_7_MK7, or ^2^H_7_MK9 for one week (Supplemental Figure 1B). Although MK7 was not included in Study 1, ^2^H_7_MK7 was added in Study 2 as a relevant dietary form due to increased clinical interest of MK7 as a dietary supplement.^39,40^ Mice were 19 weeks of age at nonfasted sacrifice, at which time cecal contents and feces were collected.

### In vitro study (study 3)

Stool samples were obtained from five healthy donors from a previous study, which was approved by the US Army Research Institute of Environmental Medicine Institutional Review Board and registered on www.clinicaltrials.gov as NCT02423551.^41^ Donors provided informed consent for their de-identified samples to be used for future research which included the present study. Donors were 23–60 yr of age, had a BMI of 18.5–30 kg/m^2^, had not taken any oral antibiotics or had a colonoscopy within the previous 3 mo, did not have any gastrointestinal disease, and did not regularly take medications affecting gastrointestinal function. Patterns of fecal MKn are associated with enterotypes, such that the *Bacteroides* enterotype is associated with fecal MK9 and MK10, and the *Prevotella* enterotype is associated with fecal MK5-MK7/MK11-MK13.^9^ We, therefore, selected donors from both major types to capture a wider MKn metabolic capacity: two donors exhibited a Prevotella-dominant enterotype, two exhibited a Bacteroides-dominant enterotype, and one donor had a more moderate Prevotella:Bacteroides ratio.

Fermentations were conducted using an HEL BioXplorer 100 system (HEL Group, Borehamwood, United Kingdom). A nutrient-rich, anaerobic fermentation medium mimicking ileal chyme, complex colonic medium (CCM),^42^ was prepared and added to bioreactor reactor vessels assembled as previously described.^43^ Bioreactors were kept at physiologic temperature under a constant flow of nitrogen to ensure anaerobicity, and pH was maintained at pH = 6.8 to mimic the conditions of the distal colon. Vitamin K or pre-vitamin K quinones (^2^H_8_MD, ^2^H_7_PK, ^2^H_7_MK4, ^2^H_7_MK9) were solubilized in Tween-80/acetone mixture, dried down under nitrogen, and then reconstituted in 5.0 mL water prior to addition to fermentation vessels. Vitamin K concentrations were targeted to a final concentration of 5 uM (5000 pmol/mL) in media. Controls included a vessel inoculated with stool but no vitamin K (“cell control”), an inoculated vessel with no vitamin K but added Tween-80 (“vehicle control”), and a vessel with all the supplemented VK quinones but no inocula to account for potential losses of vitamin K over the course of the experiment unrelated to bacterial metabolism (“photooxidation control”) (Supplemental Figure 1c).

Prepared VK was added to fermentation vessels and allowed to equilibrate prior to the addition of the pooled stool sample; the addition of the 20% fecal slurry marked T = 0 h. Aliquots were taken at 0,5,10,24, and 48 h, and immediately centrifuged to separate into pellet and supernatant fractions. For VK measurements, both fractions were analyzed at all timepoints. In preparation for 16S sequencing, pellet fractions were resuspended in glycerol phosphate buffer to be stored for later extraction (0 h and 24 h only).

The experiment was conducted in triplicate, therefore n = 3 for all vessels at all timepoints with the following exceptions: ^2^H_7_MK4 vessel had only n = 2 at 48 h, as the bioreactor lost pH control during second experiment after 24 h, and the vehicle control had n = 2 for all timepoints also due to mechanical failure.

### Vitamin K analysis

Vitamin K quinones were extracted and chemically detected by LC-MS as previously described,^44^ with the following modifications. The assay was adapted to additionally select for ^13^C_6_-labeled vitamin K forms (all unlabeled VK form m/z + 6), or ^2^H_7_-labeled forms (all m/z + 7), and K_1,25_ (m/z 521.6) used as an internal standard.

The LC-MS method was additionally adapted and abbreviated for a C30 column (ProntolSil, 5 μm, 250 mm × mm) based on an existing C30 method^45^ to remove interference to measure stable isotope-labeled (^2^H_7_- or ^13^C_6_-) MK4 in mouse feces or fecal culture aliquots.

### Vitamin K statistical analyses

Prior to analysis, nondetectable (ND) concentration values were replaced with an insignificant nonzero value below the limits of detection. Data were examined for normality and ln-transformed prior to statistical testing. In all tables, concentration data are presented as geometric means ± SEM.

VK forms within feces were analyzed by 2-way ANOVA (sex and treatment) with an interaction term. If the interaction term was nonsignificant, it was dropped from the model, but if significant analyses were stratified on sex. If diet group was significant, pairwise comparisons were conducted using Tukey’s Honestly Significant Difference (HSD). VK forms in fermentation aliquots (supernatant and pellet separately) were analyzed by repeated measures ANOVA ([lnVKform] ~ time + treatment + time*treatment). All significance testing was evaluated using two-tailed tests at an α-level of 0.05, and all analyses were done using R Version 3.6.3 (2020–02-29).

### Microbial community analyses

DNA was extracted from mouse cecal samples for Studies 1 and 2, and from starting inocula and culture aliquots for Study 3 using the MoBio PowerSoil DNA extraction kit (Study 1) or the Qiagen QIAamp PowerFecal DNA Kit (Studies 2 and 3) according to the manufacturer’s protocols. Barcoded 16S rRNA V4 region amplicon libraries were generated for each of the three studies described above and run using 250bp paired end sequencing on an Illumina MiSeq with further details as follows. For Study 1, the V4 region was amplified as previously described.^46^ Following PCR, the products were batch normalized (Invitrogen SequalPrep DNA Normalization Plates). Recovered product was pooled and cleaned using AmpureXP magnetic beads (Beckman Coulter; 0.8X beads:pool ratio) and quantified prior to loading onto an Illumina MiSeq at the Research Technology Support Facility Genomics Core at Michigan State University. For Studies 2 and 3, V4 region amplicons were generated with the described PCR primers (F515/R806).^47^ PCR reactions consisted of 5 μl HotStarTaqPlus mastermix (Qiagen), 0.25 μl 10 ml forward and reverse primers, and 25 ng template DNA or no template controls (run on each reaction mix). PCR reaction parameters were: 95°C for 5 min, 30 cycles of 95°C for 30 s, 53°C for 45 s and 72°C for 45 s, then 72°C for 10 min. PCR reactions were run in triplicate and then pooled. DNA concentrations were determined by QuantiT dsDNA assay kit (Invotrogen), and an equimolar amplicon pool was generated and purified by PCR Cleanup DNA kit (Qiagen) and Ampur Bead extraction (Agencourt). The resulting pool was then loaded onto an Illumina MiSeq at the Tufts University Core Facility.

Raw sequences were processed as amplicon sequence variants (ASVs) with the DADA2 pipeline^48^ as described^49^ (pipeline here: https://github.com/amoliverio/dada2_fiererlab). In brief, sequences were quality filtered (truncLen = 240 for forward and 160 for reverse reads, maxEE = 2, and truncQ = 2). Sequence variants were inferred with the DADA2 algorithm, and then we merged paired-end reads. Chimeras were filtered and runs were merged prior to taxonomic assignment as per the DADA2 pipeline, using the SILVA database v132^50^ using the RDP naïve Bayesian classifier.^51^ We filtered the table to remove chloroplast, mitochondrial reads and likewise removed reads that were unassigned at the phylum level. We rarefied the three datasets, respectively, to: 11,320 reads per sample for Study 1 (91 samples retained for downstream analyses), 44,455 reads per sample for Study 2 (69 samples retained), and 122,177 reads per sample for Study 3 (39 samples retained).

Microbial communities were visualized by NDMS ordination, and differences in bacterial composition were evaluated using PERMANOVA (by sex and diet in animal studies, and vitamin K treatment and timepoint in fermentation studies). *Post hoc* (Tukey HSD) comparisons were conducted if a main effect was significant (*p* < .05), and pairwise comparisons *p*-values were FDR-corrected (and significance evaluated at an FDR-corrected *p*-value of 0.05). Species diversity was assessed with the Shannon diversity index. Kruskall-Wallis tests were used to assess enrichment of specific taxa across diet groups. *P*-values were FDR-corrected for enrichment, but *post hoc* pairwise testing (Tukey HSD) *p*-values were not to allow for hypothesis generation. All statistical analyses were executed with R (versions 3.5.1–3.6.3) within the RStudio environment.

## Supplementary Material

Supplemental Material

## Data Availability

The 16S sequencing data used in this article are available in the NCBI repository under BioProject ID PRJNA669541: Influence of vitamin K on gut microbiota.

## Disclaimers

The authors declare that they have no conflicts of interest with the contents of this article. Any opinions, findings, conclusions, or recommendations expressed in this publication are those of the authors and do not necessarily reflect the views of the USDA, the US Army, or the US Department of Defense. Any citations of commercial organizations and trade names in this report do not constitute an official Department of the Army endorsement or approval of the products or services of these organizations.
